# Supplementary material for: Synovial Fluid-Derived Extracellular Vesicles of Patients with Arthritides Contribute to Hippocampal Synaptic Dysfunctions and Increase with Mood Disorders Severity in Humans
Source: Cells. 2022 Jul 23;11(15):2276. doi: 10.3390/cells11152276 (PMC9331474; doi:10.3390/cells11152276)
Supplement: Supplementary file 1 [file cells-11-02276-s001.zip › cells-1776069-supplementary.pdf]

Supplementary Materials

Suppl Figure 1

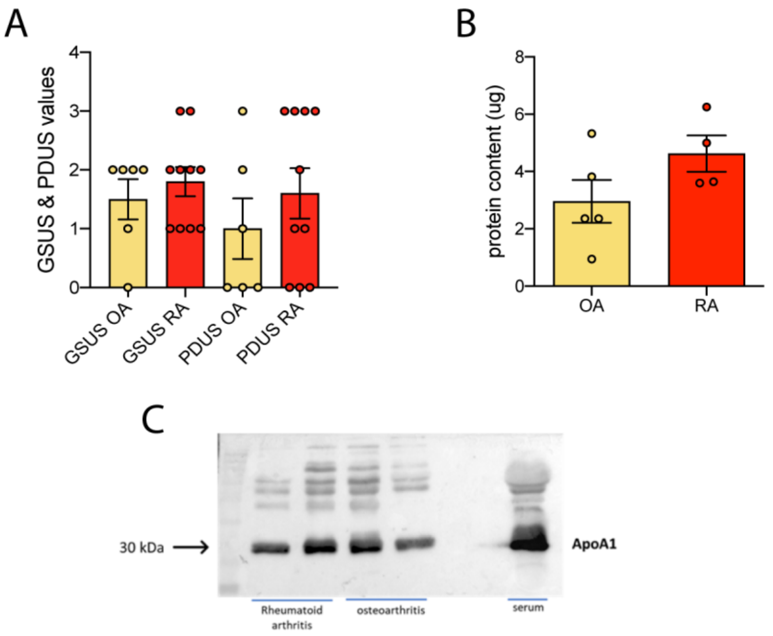

Figure S1. (A) PDUS and GSUS values of OA- and RA-patients indicate comparable level of inflammation between the two groups. (**B**) Protein content detected by micro BCA protein assay is higher in RA- respect to OA-extracted EVs, even if it is not statistically significant (Mann Whitney test, ns). **(C)**

Suppl Figure 2

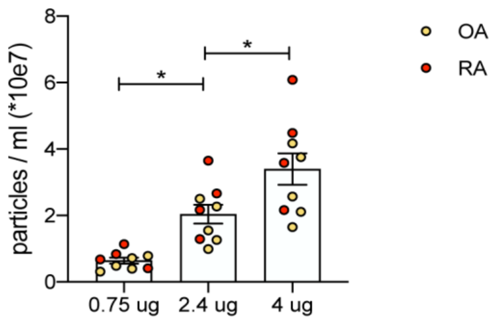

Figure S2. Graph representing the total amount of EVs corresponding to 0.75 - 2.4 - 4 µg of protein; coherently, the number of particles / ml increases as the concentration is augmented. (Ordinary one-way ANOVA followed by Tukey's multiple comparison test, ****p<0,0001; 0.75 µg=9, 2.4 µg=9, 4 µg=9). OA data are presented in yellow; RA data are presented in red.
